# Editorial Notes

**Published:** 1909-06

**Authors:** 


### Editorial Notes.

**The University of Bristol.**

THE long-continued efforts of many pioneers in the movement for obtaining University honours for Bristol and the West of England have at length met with well-deserved success—the Charter of the University of Bristol is signed, sealed and delivered. It was inevitable that, in a cause which demanded a large measure of faith and tenacity in the face of years of discouragement, many of these early workers should have passed from our midst, leaving us with a sense of deep regret that they cannot share in the city's legitimate sense of triumph; but a goodly number are with us yet to aid with their matured judgment in the early growth and development of the youngest British University. The names of those who, by the offices they have worthily filled on the University College Council and staff, or in generously giving towards the necessary funds have fostered the University movement, have been the subject of previous notices in our pages. To them all honour. But we may, perhaps, offer special congratulations to the Lord Bishop of Hereford and Mr. J. W. Arrowsmith—two of those who are pioneers in the truest sense. One of the foremost educationists of his day, and endowed with quite exceptional powers of organisation in such matters, the Bishop of Hereford gave a great impetus to the University College movement in Bristol that he had largely initiated; and Mr. Arrowsmith has worked with characteristic energy and whole-hearted devotion to the cause, and has succeeded, Prometheus-like, in bringing us fire from heaven.

**R.A.M.C.**

BRISTOL medical men, as well as the citizens of this city, have reason to be proud of the success that had attended the formation of the 3rd South Midland Field Ambulance, for within a year not only was the corps up to, or rather above, its establishment, but it is the first in the United Kingdom to reach this position except one unit that had a Bearer Company to start with as a nucleus. That this has entailed a very arduous amount of work will well be understood. It involved the examination of every recruit, the instruction of most of the recruits in the very elements of infantry drill and of first aid work, besides the more advanced training in field service work and stretcher drill. Such is the composition of a field unit, that the corps finds numerous outlets for energy, for the management and care of horses is needed as well as proficiency in driving and riding. When to all this there is added the vast correspondence, office details, preparations and working of camp and pay accounts, it will readily be understood that the duties of the commanding officer are no sinecure.

The corps has been much hampered in many ways by the absence of head-quarters of its own. For the past year or more they have been housed in the drill hall of the 4th Glo'sters, two small rooms being allotted to it for orderly room and quartermaster's store, and the use of two more on certain nights. At the present moment a large quantity of necessary stores are waiting to be sent simply because there is no room anywhere to store the big articles, such as wagons, water-cart and tents. It is satisfactory to learn that this will soon be remedied.

Last August Lt.-Col. A. W. Prichard took about 100 men to Swanage and Salisbury Plain with seven officers. This year's camp will be at Lyndhurst, and will be attended by a full strength of officers (10) and men (220); indeed it may have more than its strength in men, as under certain conditions and for certain duties the corps can recruit above its establishment. The value of camp life and discipline cannot be over-estimated, and was conspicuous by the great strides made by all who attended.

On May 23rd, Empire Sunday, a church parade was held at Redcliff with the Gloucestershire Engineers. About 250 were on parade, and looked extremely smart and soldierly in the blue walking-out dress. The band—for the corps has its own band, both brass and bugle—gives additional smartness, and is a great asset when parades take place.

The subscription list which was opened in April, under the auspices of the Bristol Medico-Chirurgical Society, to make a presentation to the corps, has been well responded to, and already amounts to £10 10s. 6d. It is hoped that further subscriptions will soon raise the total to the required sum of £15 15s. The presentation will take the form of a Drum-Major's Staff, and arrangements are being made to formally present this mark of appreciation at a parade in June.

The officers are Lt.-Col. A. W. Prichard, V.D., in command, Major James Young, Captain B. M. H. Rogers, Lieutenants J. Smith Mather, J. A. Green, P. Moxey, C. Lavington, and Quartermaster and Hon. Lieut. H. Lambert, while M. C. Coleridge and C. Corfield are only waiting for their commissions to appear in the *Gazette*. The establishment of officers is thus complete. It may be said without fear of contradiction that the present satisfactory state of the corps is due to the regular attendance of the officers at drills, and ungrudging hard work that has been put in by one and all of them to make the men efficient in drill, first aid and transport work. Five drills a week has been the rule, so that if the men do not know their work it is their own fault, and the blame cannot be laid on the officers.

**Electrical Methods of treatment.**

The paper by Dr. Herbert Tayler on "Electricity in General Practice" (page 145), and the further papers on "Ionisation or Cataphoresis," by Dr. H. P. Tayler and Dr. Lewis Jones (page 132), give us an opportunity of directing especial attention to a subject which has until recently been very disappointing in medical treatment, and has only persistently been carried out by a few enthusiastic specialists. Of late years the utility of the X-rays, and the more general adaptation of electrical methods to clinical work has opened up an entirely new field, and has led to developments which appear to have no limit. The subject of treatment by ionisation is obviously in its infancy, but already its field of utility is so wide that it demands the most careful attention of all who are engaged in the work of practical medicine. The papers we now publish show how wide this field already is, and will be quite a revelation to those who have as yet not studied the subject. We are indebted to Dr. Lewis Jones for the initiation of much of this work, but his pupils are spreading and advancing the methods with great zeal and industry. We think that his presence at a recent meeting of this Society will stimulate the use of ionic medication in this district.

**The Campaign against Tuberculosis.** THE International Congress at Washington, held last year, has given a great impetus to the work of the National Association for the Study and Prevention of Tuberculosis in the United States. A volume issued by the New York Charities' Publication Committee (1908), through the generous co-operation of the Russell Sage Foundation, gives a summary of what has been done, and appends a directory of Institutions dealing with Tuberculosis in the United States and Canada. Since the foundation of the first of these, the Adirondack Cottage Sanatorium, by Dr. Edward L. Trudeau, in 1885, the growth of the movement has been so rapid that these institutions have sprung up in all parts, so that there are now 250 special sanatoria, and new ones are announced almost daily. The promise of restoration of patients to wage-earning capacity has appealed with effective force to legislators as well as to private philanthropy. Recently the dispensary has come into prominence, but the provision for advanced and hopeless cases remains the distressing lack in our equipment, both in America and in our own country also.

The diminution in the death-rate for tuberculosis in the last two decades is well known and remarkable. Some recent evidences of this, given from statistics in Germany, have recently been published (Bandolier and Roepke, *Tuberculosis in Diagnosis and Treatment*, translated by Morland, London, 1909). In spite of this diminution, it is estimated that the number of sufferers from pulmonary tubercle in Germany is about 800,000, those capable of conveying infection about 600,000, and the number of fatal cases yearly some 80,000; hence we must, "in a chastened spirit, admit the necessity of further prophylactic and curative measures." It is again emphasised that "the whole modern campaign against this enemy of mankind is dominated by the attempt to improve the methods of diagnosis on the one hand and to make them accessible to the practitioner on the other. It stands or falls with this improvement in diagnostic method, with the early diagnosis of tuberculosis."

The annual report of the Winsley Sanatorium for 1908 shows what is being done in this district. From this it will be seen that during the past year 215 patients were discharged, and of these

89=41.4 per cent. were considered fit to return to work ;

82=37.7 per cent. improved, and many returned to work.

This gives a total of 171 (=79.1 per cent.) who had decidedly improved under treatment, and were for the most part able to resume some wage-earning occupation. The medical officer observes that " no sanatorium has yet been able to fill its beds with early cases only, but it is hoped that each succeeding year will give an increasing proportion of early cases, as it becomes more widely realised that consumptives, if sent to a sanatorium sufficiently early, can make complete recovery, and be restored to full working capacity." In spite of the excellent work being done, it is much to be regretted that this Sanatorium is still hampered by a heavy mortgage debt. The funds are, however, ample to pay all costs, if the interest on the debt could be removed.

**The Zoophil-Psychosis.**

We learn from the *Medical Record*, March 6th, 1909, that Dr. C. L. Dana, New York, has written a very useful article on this modern malady, and he give numerous illustrative cases. This psychasthenia appears to be a rapidly-spreading disease of our modern civilisation, allied to hysteria, neurasthenia and occupation psychosis of modern life. The author pleads for sick humanity, and against the excesses in such sentiment for animals as leads to selfishness and injustice, and the development of more psychopathic states. There is growing up an enormous mass of artificially cultivated tenderness towards a suppositious suffering. There will come next tears over the suffering of a fading flower, and sorrow over the unquenched thirst of the withering plant. Our anti-vivisection societies are furthering the development of this condition by mutual encouragement among the unstable and self-indulgent; whose psychosis becomes a source of distress to self and friends, and leads to demoralisation to the community culminating in serious social injustice.

---

### Notes on Preparations for the Sick.

